# Allergic contact dermatitis to corticosteroids: experience of a referral clinic from 2014 to 2018^☆^

**DOI:** 10.1016/j.abd.2020.12.012

**Published:** 2022-03-17

**Authors:** Mariana de Figueiredo Silva Hafner, Amanda Ivanchuk Lopes, Nathalie Mie Suzuki, Rosana Lazzarini

**Affiliations:** a Clínica de Dermatologia da Santa Casa de São Paulo, São Paulo, SP, Brazil; b Faculdade de Ciências Médicas da Santa Casa de São Paulo, São Paulo, SP, Brazil

Dear Editor,

The use of topical corticosteroids is common in dermatological practice. Due to their anti-inflammatory and immunomodulatory effects, they are often the drugs of choice in the treatment of dermatites. However, there is little discussion regarding the role of corticosteroids as possible triggers of an allergic process.^1^, ^2^

As they have low molecular weight and are lipophilic molecules, they have the capacity to penetrate the skin barrier in a relatively easy way, binding to carrier proteins and transforming into immunogens. These are phagocytosed and sensitize T-lymphocytes so that in subsequent contacts, they might trigger a cell hypersensitivity reaction, manifesting as allergic contact dermatitis (ACD).^2^, ^3^, ^4^

The frequency of ACD to corticosteroids varies from 0.5 to 5% in the literature.^1^, ^3^ In Brazil, there are no statistical data on the topic, which justifies the performance of the current study, which was approved by the Ethics Committee of the institution. (33471620.7.0000.5479).

Through the analysis of data from the service medical records, a total of six patients diagnosed with ACD to corticosteroids was found, two (33.3%) male and four (66.7%) female patients, representing 1% of the total cases submitted to patch tests from 2014 to 2018. The mean age of these patients was 60 years (ranging from 37 to 76 years), and the symptom period ranged from five months to 17 years, with a mean time for diagnosis of five and a half years. The long period reflects a probable difficulty in accessing a specialized dermatological service, delaying the diagnosis and resulting in greater morbidity.

According to the literature, the main risk factors for ACD to corticosteroids are the presence of previous chronic dermatoses, use of corticosteroids without medical follow-up, and genetic susceptibility.^1^ Corroborating these findings, the six patients had a history of irregular and prolonged use of topical corticosteroids, as well as previous dermatoses: psoriasis in two (33.3%), stasis dermatitis in two (33.3%), and chronic eczema of undefined etiology in two (33.3%).

Four (66.7%) of the six cases had lesions in the lower limbs, two (33.3%) in the upper limbs, one (16.7%) in the trunk, and two (33.7%) had a disseminated pattern (Figure 1, Figure 2). The typical picture, as in the present cases, is a dermatosis that is refractory to treatment with corticosteroids, characterized by eczematous lesions, which may be more evident on the edges than in the center. Moreover, there may be signs indicating chronic corticosteroid use, such as skin atrophy, rosacea, perioral and paranasal dermatitis.^1^, ^5^

**Figure 1:**
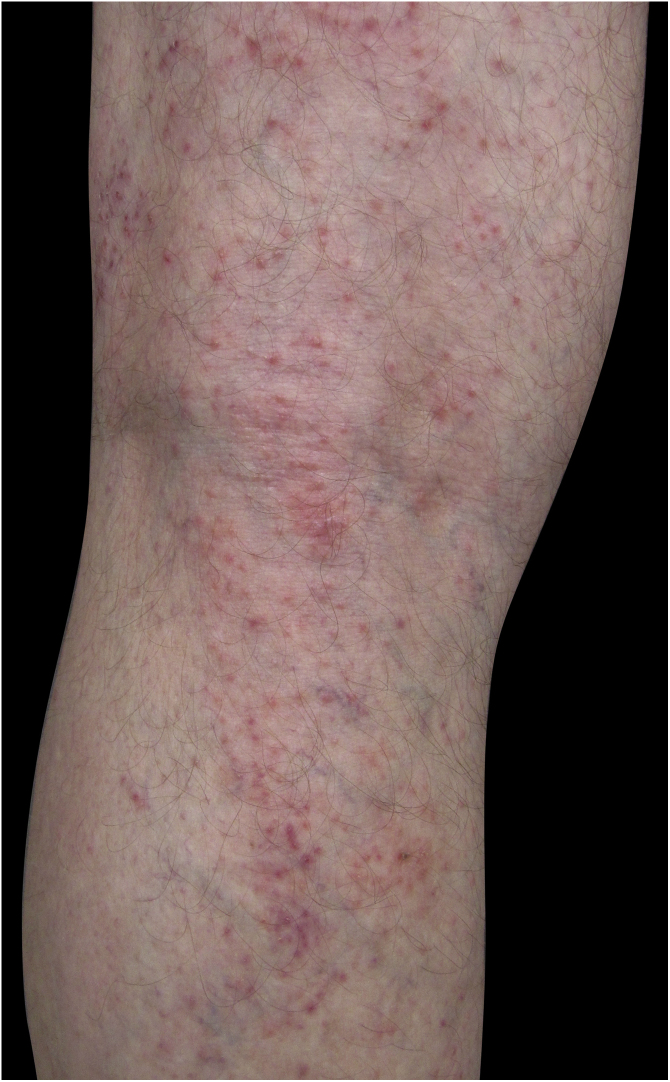
Eczema on the lower limb due to allergic contact dermatitis to corticosteroid.

**Figure 2:**
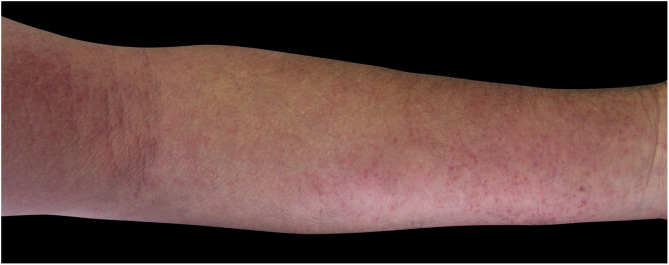
Eczema on the upper limb due to allergic contact dermatitis to corticosteroid.

In 1989, Coopman et al. categorized topical corticosteroids into four groups: A (hydrocortisone type), B (acetonide type), C (non-esterified betamethasone type), and D (esters, subdivided into D1-stable and D2-labile). In 2011, Baeck et al. proposed another classification system based on methylation patterns and allergenic profiles derived from patch tests, dividing them into three groups (Table 1). Group 1 corticosteroids more frequently result in allergic reactions, while those in Group 3 have the least sensitizing power and risk of cross-reactions. However, it is important to emphasize that the same patient may manifest hypersensitivity to more than one group.^1^, ^6^, ^7^, ^8^

**Table 1:** Classiﬁcation of the corticosteroids (Baeck et al., 2011).^7^

Group 1	Group 2	Group 3
Non-methylated and mostly non-halogenated (Group A and D2 of the Coopman classification, and budesonide)	Halogenated with C16/C17 cis ketal/diol structure (Group B of the Coopman classification)	Halogenated and C16-methylated molecules (Group C and D1 of the Coopman classification)
Budesonide	Triamcinolone	Alclomethasone dipropionate
Cloprednol	Amcinonide	Beclomethasone dipropionate
Cortisone acetate	Desonide	Betamethasone
Dichlorisone acetate	Flucoronide	Betamethasone 17-valerate
Difluprednate	Flumoxonide	Betamethasone dipropionate
Fludrocortisone acetate	Flunisolide	Betamethasone sodium phosphate
Fluorometholone	Fluocinolone acetonide	Clobetasol propionate
Prednisolone acetate	Fluocinonide	Clobetasone butyrate
Hydrocortisone	Halcinonide	Deoxymethasone
Hydrocortisone aceponate	Triamcinolone acetonide	Dexamethasone
Hydrocortisone acetate	Triamcinolone benetonide	Dexamethasone acetate
Hydrocortisone 17-butyrate	Triamcinolone diacetate	Dexamethasone sodium phosphate
Hydrocortisone 21-butyrate	Triamcinolone hexacetonide	Diflucortolone valerate
Hydrocortisone succinate		Diflorasone diacetate
Isofluprednone acetate		Flumethasone pivalate
Mazipredone		Fluocortin butyl
Medrisone		Fluocortolone
Methylprednisolone acetate		Fluocortolone caprylate
Methylprednisolone aceponate		Fluocortolone pivalate
Methylprednisolone hemisuccinate		Fluocortolone acetate
Prednicarbate		Fluprednidene acetate
Prednisolone		Halomethasone
Prednisolone caproate		Meprednisone
Prednisolone pivalate		Fluticasone propionate
Prednisolone sodium metasulfobenzoate		Momethasone furoate
Prednisolone succinate		
Prednisone		
Tixocortol pivalate		

The patch test is the gold standard diagnostic tool and should be performed to confirm the suspicion and individualize the therapeutic approach, which can range from the substitution of the corticosteroid by another that belongs to a different group to complete withdrawal of this pharmacological class.^1^, ^5^

Studies have shown that tixocortol pivalate, budesonide, and hydrocortisone 17-butyrate are the corticosteroids most likely to cause ACD.^2^ This is probably due to the greater capacity of these molecules to bind to the arginine in serum proteins, forming an antigen.^1^ These markers identify the majority of patients with ACD to corticosteroids; however, since they all belong to Group 1, patients who are allergic to the other groups may go unnoticed. Therefore, in patients who are positive for any of these markers and in those with high clinical suspicion, the ideal action is to expand the patch test study by using a specific series of corticosteroids containing drugs from all groups.

Patients with ACD to corticosteroids diagnosed in the service where the present study was carried out were tested with the Latin American supplementary battery (Chemotechnique), which contains hydrocortisone 17-butyrate, budesonide, and tixocortol pivalate, in addition to the standard Brazilian battery (FDA Allergenic). Among them, one (16.7%) was positive only for tixocortol pivalate (Fig. 3), three (50%) were positive for budesonide, and the other two (33.3%) were positive for both budesonide and tixocortol. The use of the supplementary battery in the patch tests was essential to allow the diagnosis to be attained since there are no corticosteroid markers in the standard battery. In all cases, it was recommended to withdraw contact with corticosteroids from Group 1. Although the ideal approach would be to perform a new test with a specific battery of corticosteroids, it was not possible in these cases. In one of the patients with disseminated lesions, methotrexate and phototherapy were used. The others showed a good response with the change of medication to Group 3 corticosteroids, such as fluticasone propionate and betamethasone valerate.

**Figure 3:**
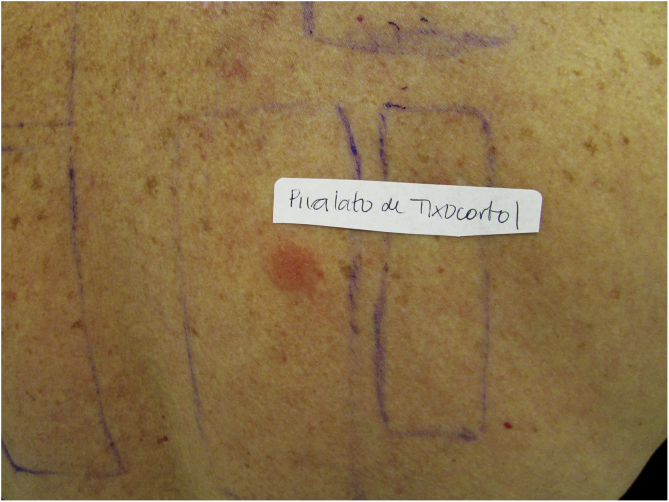
Positive patch test for tixocortol pivalate.

Moreover, five (83.3%) of the patients were polysensitized; that is, they had three or more positive reactions to unrelated allergens. These allergens were components of topical medications (neomycin, bacitracin, benzocaine, promethazine), additives (Kathon CG®, formaldehyde, thimerosal), rubber components (thiuram and carba-mix), and metals (nickel sulfate, cobalt chloride), data compatible with the literature, as these are individuals with chronic dermatoses, who have a breach in the skin barrier, and are sensitized to multiple components of medications, emollients, and other contact agents.^1^, ^9^, ^10^

There are also other elements present in topical medications (in addition to the active ingredient) that can act as allergens, such as stabilizers (e.g., ethylenediamine), vehicles (e.g., propylene glycol), and fragrances, further reinforcing the importance of patch tests.

Therefore, ACD to corticosteroids should be considered as a hypothesis in the presence of recurrent conditions, with no response to corticosteroid treatment. Patch tests should be indicated in these cases, aiming at the removal of the causal agent and the early introduction of adequate treatment.

## Financial support

None declared.

## Authors’ contributions

Mariana de Figueiredo Silva Hafner: Design and planning of the study; drafting and editing of the manuscript; collection, analysis, and interpretation of data; effective participation in research orientation; intellectual participation in the propaedeutic and/or therapeutic conduct of the studied cases; critical review of the literature; critical review of the manuscript; approval of the final version of the manuscript.

Amanda Ivanchuk Lopes: Drafting and editing of the manuscript; collection, analysis, and interpretation of data; approval of the final version of the manuscript.

Nathalie Mie Suzuki: Effective participation in research orientation; intellectual participation in the propaedeutic and/or therapeutic conduct of the studied cases; critical review of the literature; critical review of the manuscript; approval of the final version of the manuscript.

Rosana Lazzarini: Design and planning of the study; drafting and editing of the manuscript; collection, analysis, and interpretation of data; effective participation in research orientation; intellectual participation in the propaedeutic and/or therapeutic conduct of the studied cases; critical review of the literature; critical review of the manuscript; approval of the final version of the manuscript.

## Conflicts of interest

None declared.
